# Secular reduction of excess mortality in hip fracture patients >85 years

**DOI:** 10.1186/1471-2318-13-25

**Published:** 2013-03-13

**Authors:** Trine E Finnes, Haakon E Meyer, Jan A Falch, Asle W Medhus, Tore Wentzel-Larsen, Cathrine M Lofthus

**Affiliations:** 1 Department of Internal Medicine, Innlandet Hospital Trust, Skolegata 32, Hamar, N-2318, Norway; 2 Faculty of Medicine, University of Oslo, Klaus Torgårds vei 3, Oslo, N-0372, Norway; 3 Division of Epidemiology, Norwegian Institute of Public Health, Marcus Thranes gate 6, Oslo, N-0473, Norway; 4 Department of Community Medicine, Faculty of Medicine, University of Oslo, Kirkeveien 166, Oslo, 0450, Norway; 5 Department of Medicine, Oslo University Hospital, Kirkeveien 166, Oslo, N-0450, Norway; 6 Biostatistics and Epidemiology Unit, Oslo University Hospital, Kirkeveien 166, Oslo, N-0450, Norway; 7 Centre for Child and Adolescent Mental Health, Eastern and Southern Norway, Gullhaug Torg 4B, Oslo, N-0484, Norway; 8 Norwegian Centre for Violence and Traumatic Stress Studies, Kirkeveien 166, house no 48, Oslo, N-0450, Norway

## Abstract

**Background:**

More than 20% of the hip fracture patients die within the first year after the incident. Few data are available on the trends in mortality following a hip fracture. The present aim was to study changes in excess mortality after hip fracture from 1978/79 up to 1996/97.

**Methods:**

Data on 5180 hip fracture patients aged ≥ 50 years, identified in three earlier, well validated, incidence studies from Oslo were used. The studies took place in the two years periods 1978–79 and 1989–89 and in a one year period from 1st of May 1996 to 30th of April 1997. The study was designed as a historic cohort study. Exposure was sustaining a hip fracture in the registration periods. Outcome was death of all causes. Age- and sex-specific one year-mortality rates were provided by Statistics Norway. Standardized mortality ratios (SMR) were calculated for the three cohorts for each sex and age-group, for the 0–6 months, 6–12 months, 0–1 year, 1–5 years and 5–10 years intervals after fracture. To assess the duration of the excess mortality in hip fracture patients, time-framed Kaplan-Meier curves for consecutive 5-years intervals were conducted for the hip fracture patients and the corresponding background population. Only patients still alive at the start of the time interval were included. One sample log rank tests were used to test for statistical significance.

**Results:**

The one-year SMR ranged from 3.64 (2.82 – 4.61) to 4.53 (3.67 – 5.54) in men and from 2.78 (2.39 – 3.19) to 3.60 (3.19 – 4.05) in women. In the 0–6 months interval a reduction in SMR from 1978/79 to 1996/97 was observed in women aged ≥85 years. The duration of excess mortality ranged from two years in men ≥85 years to more than ten years in men and women aged 65–84 years.

**Conclusion:**

Excess mortality among hip fracture patients remains high. Over the decades, a reduced excess mortality was mainly seen in the oldest patients, suggesting that specific efforts intending to improve prevention and treatment of osteoporosis and osteoporotic fractures in the youngest elderly are required.

## Background

Hip fractures are associated with high morbidity and mortality [1-3]. Most studies over the last five decades report a one-year mortality ranging from 15-30% [4-9], and the mortality tends to be higher in men than in women [8,10]. The highest mortality is observed within the first 6 months after the fracture and declines thereafter [5,6,10].

Whereas the relative excess mortality in hip fracture patients is highest among the youngest, the absolute excess mortality is highest among the oldest [5]. The excess mortality is associated with both pre-existing comorbidity and fracture related complications e.g. infections and delirium [1,6,11,12]. Few studies on secular trends and changes in excess mortality after hip fractures over a prolonged period are available and the results are conflicting [5,8,9,11,13]. In addition, the duration of the period with excess mortality is uncertain [9,11,13].

Oslo, the capital of Norway, has the highest reported incidence of hip fracture world-wide [14]. The incidence has been reported every decade since the 1970’ies [14-16]. However, reports on mortality after hip fractures for this area are limited [6]. The present aim was to study secular trends in excess mortality after hip fracture in Oslo in the period of 1978–1997.

## Methods

### Study population

Data from three earlier incidence studies on hip fracture in Oslo were used [14-16]. These studies include all patients ≥20 years with hip fracture in the two-year periods 1978 to 1979 (n=2067) and 1988 to1989 (n=2697), and in the one-year period from 1st of May 1996 until 30th of April 1997 (n=1290). Hip fractures (International Classification of Diseases, ninth revision (ICD-9) code 820.X) were identified through diagnosis registers, operating theatres protocols, medical records, and x-ray records. Fractures in patients residing outside Oslo or fractures due to malignancy were not included in these studies.

Hip fractures in patients aged <50 years are infrequent, and the younger hip fracture patients differ considerably from the older hip fracture patients regarding comorbidity [17,18]. For this reason, patients aged <50 years were not included in the current study. For patients with more than one fracture in any of the inclusion periods, the first fracture was included in the present study.

Registration cards from the incidence studies of 1978/79 and 1988/89 were retrieved, and the cases were identified through name and date of birth. The data from the registration cards were transferred into an electronic register, and linked to the National Population Register (Norwegian Tax Administration, Oslo) to achieve the full unique Norwegian 11-digit identification number for each patient. The data from 1996/97 were stored in an electronic register with the 11-digit identification number.

In the 1978/79-cohort, 50 patients were aged <50 years. Of the patients aged ≥50 years, 80 patients were not included due to: I) unidentifiable date of fracture (n=53); II) loss to follow up (n=1); III) missing unique identification number (n=24); IV) fracture due to metastasis (n=1); V) double registration (n=1). There were no differences regarding sex, age, and type of fracture when comparing the excluded patients with the included patients (Chi-square test and Student’s *t* test).

In the 1988/89-cohort, 26 patients who had sustained a hip fracture in the previous inclusion period and 42 patients aged <50 years were not included. In addition, ten patients were not included due to: I) unidentifiable date of fracture (n=2); II) loss to follow up (n=4); III) missing unique identification number (n=3); IV) resident outside Oslo (n=1).

In the 1996/97 cohort, 15 patients had sustained a hip fracture in one of the earlier inclusion periods, and 21 patients were aged <50 years. All the remaining patients were included.

Data from the 5180 cases included (Table 1) were linked to The Cause of Death Register provided by Statistics Norway (Statistics Norway, Kongsvinger, Norway). The patients were followed up with respect to death from all causes until 31st of December 2007 (Additional file 1: Flow chart).

**Table 1:** Characteristics of the included patients of the different cohorts

	**1978/79**	**1988/89**	**1996/97**	p-value*
Included, n	1937	2619	1254	
Men, n (%)	215 (21.5)	546 (20.8)	279 (22.2)	0.597
Mean age at time of fracture, men (95% CI)	74.2 (73.3 – 75.2)	76.6 (75.9 – 77.4)	79.5 (78.4 – 80.6)	<0.001
Mean age at time of fracture, women (95% CI)	76.8 (76.4 – 77.3)	80.2 (79.8 – 80.6)	81.4 (80.9 – 82.0)	<0.001
50-64 years, n (%)	254 (13.1)	170 (6.5)	60 (4.8)	
65-84 years, n (%)	1322 (68.2)	1606 (61.3)	719 (57.3)	
≥85 years, n (%)	361 (18.6)	843 (32.2)	475 (37.9)	
Intertrochanteric fractures, n (%)	751 (38.8)	1085 (41.4)	563 (44.9)	0.003
Median follow up time, years (range)	4.9 (0–30.0)	3.37 (0–20.0)	3.63 (0–11.7)	

* p-value for differences between the cohorts.

### Study design

The study was designed as a historic cohort study. Exposure was sustaining a hip fracture in the registration periods. Outcome was death from all causes. Covariates were sex, age, and type of fracture.

### Data and definitions

The hip fractures were defined as femoral neck or intertrochanteric. Subtrochanteric fractures were not included.

Hip fractures in patients aged ≥65 years are often referred to as geriatric hip fractures, and younger patient are frequently not included in survival studies [8,13]. Those aged ≥ 85 years have a high absolute mortality during the first six months of follow up, and a shorter duration of excess mortality than younger hip fracture patients [5,7,9]. The patients were therefore divided into following age groups: I) 50–64 years; II) 65–84 years; and III) ≥ 85 years.

In the 1978/79-cohort, fracture time was only registered by month and year. Date of fracture was consequently set to the first of the month to avoid negative survival time. To assess the influence of this fictive fracture date, survival analyses with the fracture date set to both the 1st and the 15th of the month were performed. The different fictive fracture days did not influence the results presented.

### Statistical analyses

Comparisons of baseline data between the three cohorts were made using the Kruskal Wallis test and the One-Way-ANOVA. Kaplan-Meier-curves were estimated for each cohort stratified by sex, age group, and fracture type.

**Figure 1:**
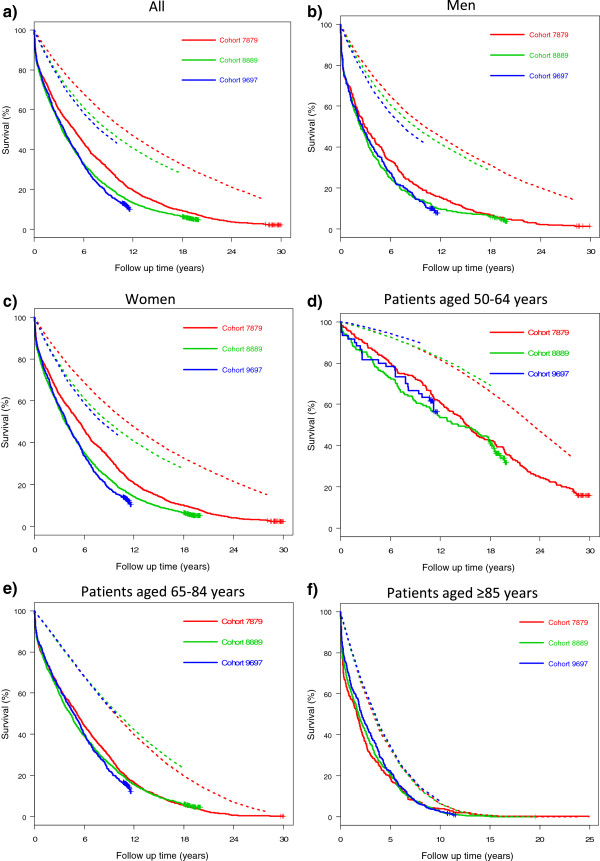
**Expected survival and observed survival by cohort.** Expected survival (dotted curves) and observed survival (continuous curves) by cohort. **a**) All, **b**) Men, **c**) Women, **d**) Patients aged 50–64 years, **e**) Patients aged 65–84 years, and **f**) Patients aged ≥85 years (Please note slight difference in the time scale). A substantially higher mortality in hip fracture patients, than in the corresponding background population, is shown. The better survival in the 1978–79 cohort in the analyses not stratified on age is explained by the younger patients in this cohort.

Age- and sex-specific one year-mortality rates for Oslo from 1978–2007 were provided by Statistics Norway and were used to calculate the expected survival curves for each cohort (Figure 1) as described by Therneau [19].

Standardized mortality ratio (SMR) expresses the level of excess mortality [20], and was calculated as the ratio of patient mortality to mortality in the background population of Oslo. The background population corresponded to the study population with respect to time period, sex, and year of birth. SMRs were calculated for the three cohorts for each sex, age-group, and fracture type, for the 0–6 months, 6–12 months, 0–1 year, 1–5 years and 5–10 years intervals after fracture. Sex- and age stratified analyses were also performed for the 0–1 year interval to allow comparison with earlier studies. Confidence intervals for SMR were computed as bootstrap BC_a_ intervals with 10 000 replications [21].

To assess the duration of the excess mortality, time-framed Kaplan-Meier curves for 5-years intervals starting at each year of the follow up time were calculated for each sex and age-group. These curves were compared with the corresponding expected survival curves. Only patients still alive at the start of the time interval were included. One sample log rank tests were used to test for statistical significance between observed and expected curves. The beginning of the last 5 year interval, where there still was statistical significance between the expected and observed curves, was set as duration of excess mortality.

The level of statistical significance was set at p<0.05. Analyses were performed using SPSS 14.0 (SPSS Inc., Chicago, IL, USA) and R 2.10.1 (The R Foundation for Statistical Computing, Vienna, Austria) with the R packages survival and boot.

### Ethics and approvals

The study is performed in compliance with the Helsinki Declaration. The Regional Committee for Research Ethics approved the study. A dispensation from professional secrecy was given by the Norwegian Directorate for Health and Social Affairs. Permission to handle sensitive information were sought and received from the Data Protection Agency.

## Results

### Patient characteristics

The proportion of men was 21-22% in all three cohorts (Table 1). Mean age at time of fracture increased by about 5 years in both men and women from 1978/79 to 1996/97. The proportion of patients aged ≥ 85 years increased from 19 to 38%. The proportion of intertrochanteric fractures was higher in the 1996/97-cohort than in the 1978/79-cohort (p=0.003).

### Excess mortality within each cohort

Kaplan-Meier curves of the three cohorts showed a substantially higher mortality for both sexes, in all age groups, than did the expected survival curves (Figure 1).

One-year mortality in the different cohorts ranged from 30% to 33% in men, and from 21% to 25% in women (data not shown).

The highest SMR was observed within 6 months after the fracture (Tables 2, 3, 4).

**Table 2:** Standardized mortality rates in given time intervals with respect to sex and cohort

**Follow up time**	**Sex**	**Cohort**	**n**	**Dead, n (%)**	**Expected dead, n**	**SMR (95% CI)**
0-6 months	Men	78/79	417	105 (25.2)	15.41	6.81 (4.47 – 8.35)
		88/89	546	137 (25.1)	22.96	5.97 (4.90 – 7.13)
		96/97	279	72 (25.8)	14.00	5.14 (3.90 – 6.61)
	Women					
		78/79	1520	249 (16.4)	48.74	5.11 (4.44 – 5.80)
		88/89	2073	371 (17.9)	89.67	4.14 (3.68 – 4.61)
		96/97	975	156 (16.0)	43.36	3.60 (2.99 – 4.26)
6-12 months	Men					
		78/79	312	21 (6.7)	12.41	1.69 (1.02 – 2.48)
		88/89	409	38 (9.3)	18.80	2.02 (1.38 – 2.70)
		96/97	207	19 (9.2)	11.03	1.72 (1.00 – 2.59)
	Women					
		78/79	1271	76 (6.0)	41.45	1.83 (1.42 – 2.28)
		88/89	1702	148 (8.7)	75.42	1.96 (1.65 – 2.30)
		96/97	819	70 (8.5)	38.19	1.83 (1.42 – 2.29)
1-5 years	Men					
		78/79	291	129 (44.3)	71.90	1.79 (1.47 - 2.18 )
		88/89	371	208 (56.0)	97.95	2.12 (1.82 – 2.47)
		96/97	188	95 (50.5)	60.43	1.57 (1.26 – 1.96)
	Women					
		78/79	1195	405 (33.9)	285.68	1.42 (1.27 - 1.57)
		88/89	1554	701 (45.1)	472.31	1.48 (1.37 - 1.61)
		96/97	749	342 (45.7)	245.99	1.39 (1.24 - 1.55)
5-10 years	Men					
		78/79	162	83 (51.2)	54.17	1.53 (1.14 – 2..04)
		88/89	163	88 (54.0)	54.77	1.61 (1.25 – 2..06)
		96/97	93	58 (62.3)	33.56	1.73 (1.32 – 2.26)
	Women					
		78/79	790	366 (46.3)	255.16	1.43 (1.28 – 1.60)
		88/89	853	455 (53.3)	318.87	1.42 (1.29 – 1.58)
		96/97	407	258 (63.3)	146.69	1.76 (1.54 – 2.02)

**Table 3:** Standardized mortality rates in given time intervals with respect to age group and cohort (both sexes)

**Follow up time**	**Age-group, years**	**Cohort**	**n**	**Dead, n (%)**	**Expected dead, n**	**SMR (95% CI)**
0-6 months	50-64					
		78/79	254	7 (2.7)	1.3	5.25 (1.62 – 9.57)
		88/89	170	10 (5.9)	1.0	10.54 (4.53 – 17.86)
		96/97	60	4 (6.7)	0.2	17.19 (3.66 – 37.53)
	65-84					
		78/79	1322	223 (16.9)	35.2	6.33 (5.49 – 7.20)
		88/89	1606	257 (16.0)	40.9	6.29 (5.49 - 7.15)
		96/97	719	107 (14.9)	17.8	6.03 (4.88 – 7.28)
	≥ 85					
		78/79	361	124 (34.3)	27.6	4.49 (3.67 – 5.38)
		88/89	843	241 (28.6)	70.8	3.40 (2.94 – 3.90)
		96/97	475	117 (24.6)	39.4	2.97 (2.43 – 3.60)
6-12 months	50-64					
		78/79	247	4 (1.3)	1.33	2.99 (0.68 – 16.25)
		88/89	160	3 (1.9)	0.90	3.35 (0.00 -7.86)
		96/97	56	1 (1.8)	0.22	4.54 (0.00-16.35)
	65-84					
		78/79	1099	64 (30.1)	30.14	2.12 (1.62 – 2.67)
		88/89	1349	102 (7.6)	36.64	2.78 (2.26 -3.33 )
		96/97	612	39 (6.4)	16.03	2.43 (1.70 -3.24 )
	≥ 85					
		78/79	237	29 (12.2)	22.39	1.30 (0.85- 1.81)
		88/89	602	81 (13.5)	56.68	1.43 (1.13 – 1.76)
		96/97	358	49 (13.7)	32.97	1.49 (1.09 – 1.93)
1-5 years	50-64					
		78/79	243	31 (12.8)	11.79	2.63 (1.75 -3.59 )
		88/89	157	28 (17.8)	7.41	3.78 (2.46 – 5.31)
		96/97	55	7 (12.7)	2.03	3.45 (1.12 – 6.47)
	65-84					
		78/79	1035	363 (35.1)	224.54	1.62 (1.45 – 1.80)
		88/89	1247	533 (42.7)	260.07	2.05 (1.87 - 2.24)
		96/97	573	226 (39.4)	120.21	1.88 (1.64 – 2.15)
	≥ 85					
		78/79	208	140 (0.67)	121.25	1.36 (0.97 – 1.37)
		88/89	521	348 (66.8)	302.79	1.15 (1.03 – 1.28)
		96/97	309	204 (66.0)	184.18	1.11 (0.97 - 1.27)
5-10 years	50-64					
		78/79	212	36 (17.0)	17.94	2.01 (1.38 – 2.74)
		88/89	129	28 (21.7)	9.99	2.80 (1.81 – 3.93)
		96/97	48	10 (20.8)	2.91	3.44 (1.51 – 5.91)
	65-84					
		78/79	672	359 (53.4)	233.11	1.54 (1.38 – 1.72)
		88/89	714	363 (50.8)	233.4	1.55 (1.40 – 1.73)
		96/97	347	213 (61.4)	106.23	2.01 (1.74 – 2.31)
	≥ 85					
		78/79	68	54 (79.4)	58.29	0.93 (0.66 – 1.32)
		88/89	173	152 (87.9)	130.22	1.17 (0.98 – 1.38)
		96/97	105	93 (88.6)	71.11	1.31 (1.07 – 1.61)

**Table 4:** Standardized mortality rates in given time intervals with respect to fracture type and cohort (both sexes)

**Follow up time**	**Fracture type**	**Cohort**	**n**	**Dead, n (%)**	**Expected dead, n**	**SMR (95% CI)**
0-6 months	Femoral neck					
		78/79	1185	203 (17.1)	36.7	5.53 (4.76 – 6.37)
		88/89	1534	263 (17.1)	62.6	4.20 (3.66 – 4.76)
		96/97	691	127 (18.4)	30.2	4.21 (3.44 – 5.05)
	Inter-trochanteric					
		78/79	751	151 (20.1)	27.4	5.51 (4.61 – 6.52)
		88/89	1085	245 (22.6)	50.0	4.90 (4.23 – 5.63)
		96/97	563	101 (17.9)	27.2	3.71 (2.95 – 4.56)
6-12 months	Femoral neck					
		78/79	982	61 (6.2)	30.55	2.00 (1.50 – 2..52)
		88/89	1271	105 (8.3)	51.89	2.02 (1.64 – 2.43)
		96/97	564	46 (8.2)	25.53	1.80 (1.30 – 2.36)
	Inter-trochanteric					
		78/79	600	36 (6.0)	23.31	1.54 (1.05 – 2.10)
		88/89	840	81 (9.6)	42.33	1.91 (1.51 – 2.36)
		96/97	462	43 (9.3)	23.69	1.82 (1.29 – 2.41)
1-5 years	Femoral neck					
		78/79	921	312 (33.9)	205.26	1.52 (1.32 – 1.71)
		88/89	1166	527 (45.2)	325.74	1.62 (1.47 – 1.77)
		96/97	518	224 (43.2)	161.04	1.39 (1.21 – 1.60)
	Inter-trochanteric					
		78/79	564	221 (39.2)	152.31	1.45 (1.25 – 1.67)
		88/89	759	382 (50.3)	244.52	1.56 (1.39 – 1.75)
		96/97	419	213 (50.8)	145.39	1.47 (1.27 – 1.69)
5-10 years	Femoral neck					
		78/79	609	260 (42.7)	191.54	1.36 (1.19 – 1.54)
		88/89	639	322 (50.4)	229.64	1.40 (1.25 – 1.57)
		96/97	294	176 (59.9)	99.77	1.76 (1.52 – 2.07)
	Inter-trochanteric					
		78/79	343	189 (55.1)	117.78	1.60 (1.35 – 1.90)
		88/89	377	221 (58.6)	144.01	1.53 (1.32 – 1.79)
		96/97	206	140 (68.0)	80.48	1.74 (1.45 – 2.10)

Men had higher SMR than women during all follow up intervals and in all three cohorts.

The SMRs were higher in those aged 65–84 years than in those aged ≥85 years during all follow up intervals and in all three cohorts (Table 3). There were no substantial differences in SMRs of femoral neck and intertrochanteric fractures within the cohorts (Table 4). Sex stratified analyses for each age group demonstrated higher SMR in men than in women in patients aged 56–84 and ≥ 85 years (Table 5). The lowest SMR was seen in the oldest age group in both sexes.

**Table 5:** Standardized mortality rates for the 0–1 year interval after fracture with respect to sex and cohort

	**Age-group, years**	**Cohort**	**n**	**Dead, n (%)**	**Expected dead, n**	**SMR (95% CI)**
Men	50-64					
		78/79	74	3 (4.1)	1.26	2.38 (0.00 - 5.58)
		88/89	63	5 (7.9)	0.99	5.05 (1.02 - 10.42)
		96/97	22	2 (9.1)	0.23	8.76 (0.00 - 26.70)
	65-84					
		78/79	281	93 (33.1)	17.13	5.43 (4.30 - 6.74)
		88/89	363	117 (32.2)	22.19	5.27 (4.27 - 6.41)
		96/97	170	43 (25.3)	10.92	3.94 (2.76 - 5.29)
	≥ 85					
		78/79	62	30 (48.4)	3.18	3.18 (2.00 – 4.93)
		88/89	120	53 (44.2)	2.85	2.85 (2.07 – 3.84)
		96/97	87	46 (52.9)	3.31	3.31 (2.30 – 4.70)
Women	50-64					
		78/79	180	8 (4.4)	1.41	5.67 (2.10 – 10.02)
		88/89	107	8 (7.5)	0.85	9.36 (3.42 – 16.76)
		96/97	38	3 (7.9)	0.22	13.37 (0.00 – 32.24)
	65-84					
		78/79	1041	194 (18.6)	48.22	4.02 (3.45 – 4.64)
		88/89	1243	242 (19.5)	55.33	4.37 (3.82 – 4.97)
		96/97	549	103 (18.8)	22.86	4.51 (3.62 – 5.48)
	≥ 85					
		78/79	299	123 (41.1)	40.56	3.03 (2.46 – 3.68)
		88/89	723	269 (37.2)	108.90	2.47 (2.16 – 2.82)
		96/97	388	120 (30.9)	58.47	2.05 (1.69 – 2.46)

### Secular changes in excess mortality from 1978 to 1997

Changes in SMRs over the decades were more evident during the 0–6 month interval, than during long term follow up (Tables 2, 3, 4).

In women, there was a reduction in SMR from 1978/79 to 1996/97 during the 0–6 month interval. In men, a similar trend was observed (Table 2). In the age stratified analyses a statistically significant reduction in SMR from 1978/79 to 1996/97 was evident in the 0–6 months interval in those aged ≥85 years (Table 3). Further age- and sex stratified analyses performed for the 0–1 year interval revealed a statistically significant reduction in SMR only in women aged ≥85 years and a trend towards declining SMR in men aged 65–84 years (Table 5).

The changes in SMR from 1978/79 to 1996/97 were similar for femoral neck and intertrochanteric fractures (Table 4).

### Duration of excess mortality

The longest duration of excess mortality was found in women aged 65–84 years (Table 6), where the excess mortality lasted more than 19 years in the 1978/79-cohort, for 14 years in the 1988/89-cohort, and until end of follow up in the 1996/97-cohort. The shortest period with excess mortality was seen in men aged ≥85 years. In this age-group, the mortality returned to the level of the background population after two to four years. Except for women aged ≥85 years, there was a trend towards shorter duration of excess mortality from 1978/79 to 1996/97.

**Table 6:** Duration of excess mortality according to sex, age-group, and cohort

**Sex**	**Age-group, years**	**Cohort**	**Included, n**	**Duration of excess mortality, years**
Men				
	50-64	78/79	74	6
		88/89	63	9
		96/97	22	3
	65-84	78/79	281	15 ^a)^
		88/89	363	11
		96/97	170	>10 ^b)^
	≥ 85	78/79	62	3
		88/89	120	3
		96/97	87	2
Women	50-64	78/79	180	17
		88/89	107	12
		96/97	38	9
	65-84	78/79	1041	>19 ^a)^
		88/89	1243	14
		96/97	549	10
	≥ 85	78/79	299	3
		88/89	723	7
		96/97	388	8

^a)^ Further analysis not possible due to few patients left.

^b)^ End of follow up at 10.7 years.

## Discussion

The present study demonstrates considerable excess mortality for a prolonged period after hip fracture. In the period from 1978/79 to 1996/97, excess mortality during the first six months after the fracture declined among female hip fracture patients aged ≥ 85 years. In other age groups, no statistically significant changes were seen. The duration of excess mortality ranged from 2 years in men aged ≥ 85 years to more than 10 years in women aged 65–84 years.

### Excess mortality

The observed 1-year SMRs in the present study tended to be higher than in other similar reports. One study on hip fracture caused by falls from standing height or less in patients aged > 60 years showed a 1-year SMR of 2.18 for women and 3.17 for men [22]. Another study only including cognitively intact, ambulatory patients aged ≥65 years reported an overall 1-year SMR of 1.5 [8]. The present study includes practically all hip fracture patients aged >50 years in Oslo, including those living in nursing homes. Holvik et al. reported a one-year mortality of 46% in patients admitted from nursing homes, compared with 13.7% in patients living at home at the time of fracture [23]. In the present study, place of living is not registered in all three cohorts. However, in the 1988–89 cohort, 24% of the patients stayed in nursing home at the time of the fracture. Thus, the higher mortality among nursing home residents may have contributed to the high SMR in the current study. By also including those aged 50–65 years, average SMR is expected to increase because of the high relative excess mortality in this age group.

### Excess mortality according to age

The higher relative excess mortality among the youngest compared with the oldest is in accordance with other reports [5,7,24]. The high impact of hip fracture on relative excess mortality among the youngest is probably related to the low mortality in the corresponding population, and the higher frequency of comorbidity in young hip fracture patients compared with the background population with same age [6,18,25,26].

### Excess mortality according to sex

As in other reports, we found a higher excess mortality in men compared with women [5,8,10,27]. Higher excess mortality in men is also reported in studies adjusting for the higher rates of complications and comorbidity [6,10]. The reasons for the higher excess mortality in men remain unclear.

### Excess mortality according to time after fracture

The highest excess mortality occurred within the first six months after the fracture, in accordance with earlier reports [5,6,11]. The high mortality in this period is probably a combined effect of the trauma and comorbidity [6,11]. The lower SMR during long-term follow up is to be expected, as the impact of trauma declines with time.

### Secular changes of excess mortality

Earlier reports on changes in mortality over the decades have been conflicting. A population-based British study showed a reduction in one-year mortality from 1968 to 1983, but no reduction from 1983 to 1998 [13]. Furthermore, a Danish register study reported a slightly increased excess mortality in hip fracture patients in the period 1986 to 2001 compared with 1981 to 1985 [9]. The methods used in these studies differ from the present one, which limit the possibility for comparison of the results.

### Secular changes of excess mortality according to age

The present data showed a statistically significant reduction in the 0–6 month SMR from 1978/79 to 1996/97 in patients aged ≥ 85 years. Decreased mortality following pneumonia and myocardial infarction [28-31], which are common concomitant diseases in old hip fracture patients [12], may have contributed to the improved survival in the oldest age group. One may speculate that an increasing disease burden in hip fracture patients aged < 85 years, may be a reason for the stable SMR over time in these age groups. These speculations are supported by data from 2005 – 2009 from the Norwegian hip fracture register, which shows an increased proportion of patients with an ASA- (American Society of Anaesthesiologists) score [32] of 3 (severe systemic disease) at admittance, and fewer with an ASA score of 1 and 2 (none or mild systemic disease) [33]. It is not known when this trend started.

### Secular changes of excess mortality according to sex

Although more pronounced in women than in men, there was a similar reduction in the excess mortality for both sexes during the 0–6 months interval from 1978/79 to 1996/97 in the analyses stratified on sex (Table 2). However, during the 6–12 months and the 1–5 years interval there was a trend towards increased SMR from 1978/79 to 1988/89 and a reduction from 1988/89 to 1996/97 in men, which was not found in women. The present data do not provide any explanation for this. Changes in incidence of, and survival after diseases more common in men than in women might have influenced the outcome in men [28,34]. Furthermore, Bacon et al. found a greater improvement of survival in elderly men than in women in the period 1965 to 1993 [35]. This highlights the need for separate analyses of mortality in men and women.

### Secular changes of excess mortality according to time after fracture

The present data show that secular changes in SMR are most pronounced during short time follow up. This is to be expected, as the excess mortality is highest in the early phase after the fracture. Modifiable factors which could improve survival are time from fracture to operation [36], the use of prophylactic antibiotics [37], and early mobilisation [38].

### Secular changes of excess mortality according to fracture type

Patients suffering from intertrochanteric fractures are supposed to be frailer and have more comorbidity and a higher mortality than those with femoral neck fractures [39], and the increased proportion of intertrochanteric fractures in our study could have influenced the results. However, in the present study, the SMRs were similar for the two fracture types, implying that fracture type is not a major determinant of excess mortality. The results are in concordance with other studies that have shown that the age- adjusted mortality in patients with intertrochanteric fractures is the same as for those with femoral neck fractures [40,41].

### Secular changes of duration of excess mortality

Duration of the period with excess mortality ranged from two years to more than 19 years in the different sex- and age-groups. Hip fracture patients aged 65–84 years had, in accordance with other population based studies [5,11], the longest duration of excess mortality. This prolonged period of excess mortality may reflect the high rate of comorbid conditions also among those surviving the first months after the fracture. This is further emphasised in other studies reporting prolonged excess mortality only in patients with a high degree of comorbidity [6,11]. It is to expect that the oldest patients have the shortest period of excess mortality, as a high proportion in this age group will die during the follow up. However, the present study demonstrated an increase from 3 to 8 years of the period with excess mortality among the oldest women. Expected remaining life-time in Norwegian women aged 85 years was 5.9 years in 1996 according to the data provided from Statistics Norway, and changes in duration of excess mortality are therefore considered to be of clinical relevance. The increased duration of excess mortality may be a result of the reduced 6-months and 1-year excess mortality observed in hip fracture patients ≥85 years, which may have left a higher number of frail and sick patients for the long term follow up, contributing to excess mortality for a longer time period. Contrary, the shorter duration of excess mortality in men compared with women, may reflect the higher early mortality in men, leaving few and relatively healthy subjects for follow up.

### Limitations

Although, SMRs allow comparison of mortality in cohorts from populations with different background mortality, it may be considered as a limitation that no data on comorbidity or medication were available. Hip fracture patients are likely to have a higher frequency of comorbidity than the general population [6,11,18,25], and changes in disease burden and medication in the fracture population may have differed from that in the background population. Another limitation of the study is that data were collected for only one calendar year in 1996/97, influencing the statistical power, particularly in men and the younger patients.

The strengths of the present study are the completeness of registrations of deaths and the long follow up of hip fracture patients from validated population-based incidence studies over three decades. No major changes in diagnostics took place in the actual period. The definitions remained the same, and the data collection was equal in the three incidence studies [14-16]. No electronic diagnosis registers were available in 1978/79. However, the additional identification of the hip fractures through medical records and x-rays in all three incidence studies makes the data collection accurate and comparable in all three studies [14].

## Conclusion

To conclude, hip fracture patients have a considerably higher mortality than the background population with same age and gender. Over the decades, a statistically significant reduction of one year excess mortality was only seen in the oldest women, suggesting that specific efforts intending to improve prevention and treatment of osteoporosis and osteoporotic fractures in the youngest elderly are required. Mortality studies including more recent data with a higher proportion of patients treated with bisphosphonates and hip replacement are warranted.

## Abbreviations

SMR: Standardized mortality ratio; CI: Confidence interval

## Competing interests

None of the authors have any financial or non-financial competing interest to declare.

## Authors’ contribution

TEF contributed to acquisition of data and design of the study, performed statistical analysis and interpretation of data, and wrote the manuscript. HEM participated in interpretation of data and revising of the manuscript. JAF contributed to acquisition and interpretation of data and revising of the manuscript. AWM contributed to acquisition of data and revising of the manuscript. TWL participated in the design of the study and performed statistical analysis, and helped to draft parts of the manuscript. CML contributed to acquisition and interpretation of data, conception and design, and drafting and revising of the manuscript. All authors read and approved the final manuscript.

## Pre-publication history

The pre-publication history for this paper can be accessed here:

http://www.biomedcentral.com/1471-2318/13/25/prepub

## Supplementary Material

Additional file 1 **Excluded patients.** Flow chart illustrating exclusion of patients who were included in the original incidence studies. Click here for file
