# Supplementary figures and images for: Secular reduction of excess mortality in hip fracture patients >85 years
Source: BMC Geriatr. 2013 Mar 13;13:25. doi: 10.1186/1471-2318-13-25 (PMC3610125; doi:10.1186/1471-2318-13-25)

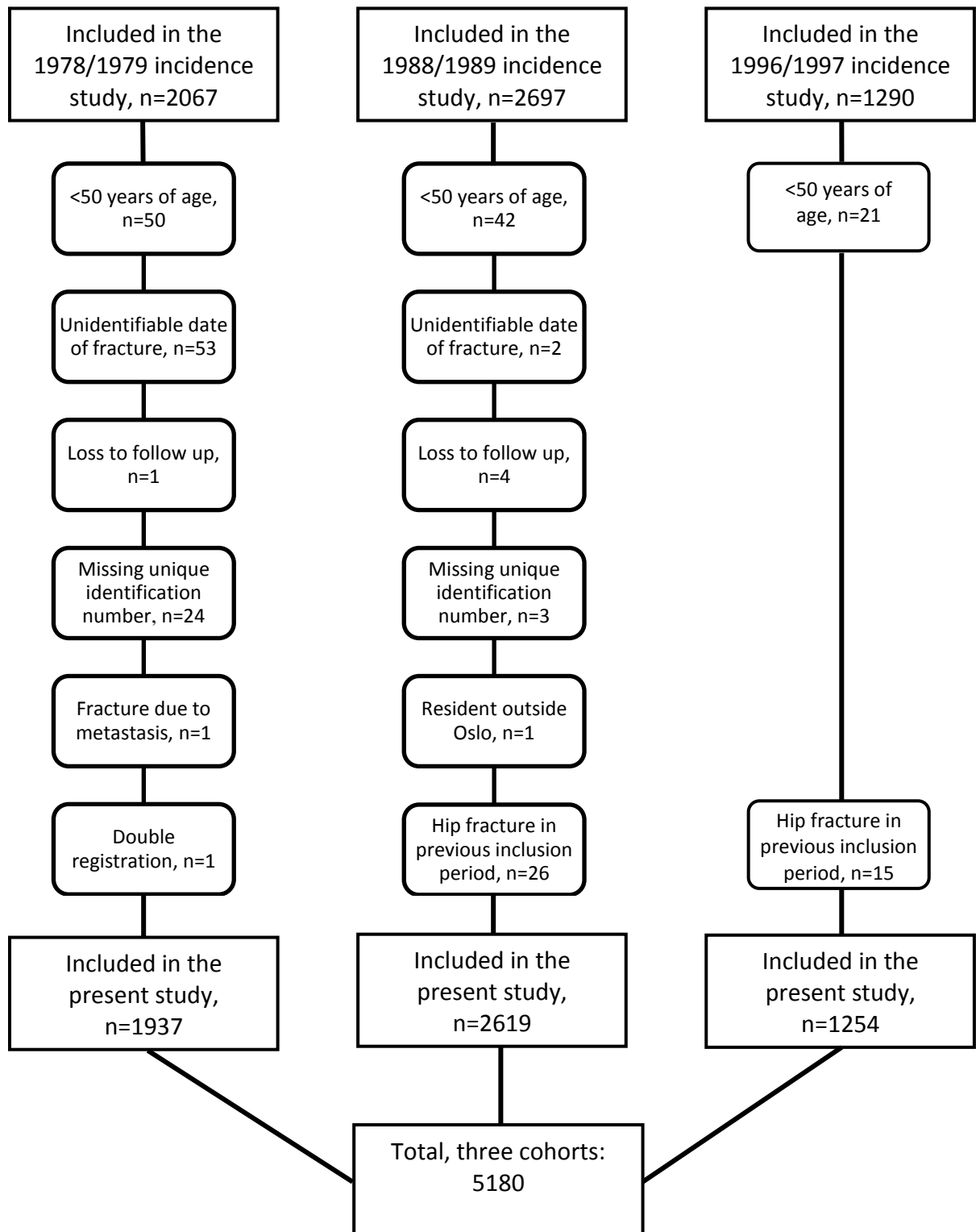

Supplement: Additional file 1 — Excluded patients. Flow chart illustrating exclusion of patients who were included in the original incidence studies. [file 1471-2318-13-25-S1.pdf]
